# Women with Multiple Chemical Sensitivity Have Increased Harm Avoidance and Reduced 5-HT_1A_ Receptor Binding Potential in the Anterior Cingulate and Amygdala

**DOI:** 10.1371/journal.pone.0054781

**Published:** 2013-01-22

**Authors:** Lena Hillert, Hristina Jovanovic, Fredrik Åhs, Ivanka Savic

**Affiliations:** 1 Department of Public Health Sciences, Division of Occupational and Environmental Medicine, Karolinska Institutet, Stockholm, Sweden; 2 Stockholm Brain Institute, Department of Women’s and Children’s Health, Karolinska Institutet, Stockholm, Sweden; 3 Center for Cognitive Neuroscience, Duke University, Durham, North Carolina, United States of America; University of Manchester, United Kingdom

## Abstract

Multiple chemical sensitivity (MCS) is a common condition, characterized by somatic distress upon exposure to odors. As in other idiopathic environmental intolerances, the underlying mechanisms are unknown. Contrary to the expectations it was recently found that persons with MCS activate the odor-processing brain regions less than controls, while their activation of the anterior cingulate cortex (ACC) is increased. The present follow-up study was designed to test the hypotheses that MCS subjects have increased harm avoidance and deviations in the serotonin system, which could render them intolerant to environmental odors. Twelve MCS and 11 control subjects, age 22–44, all working or studying females, were included in a PET study where 5-HT_1A_ receptor binding potential (BP) was assessed after bolus injection of [^11^C]WAY100635. Psychological profiles were assessed by the Temperament and Character Inventory and the Swedish universities Scales of Personality. All MCS and 12 control subjects were also tested for emotional startle modulation in an acoustic startle test. MCS subjects exhibited significantly increased harm avoidance, and anxiety compared to controls. They also had a reduced 5-HT_1A_ receptor BP in amygdala (p = 0.029), ACC (p = 0.005) (planned comparisons, significance level 0.05), and insular cortex (p = 0.003; significance level p<0.005 with Bonferroni correction), and showed an inverse correlation between degree of anxiety and the BP in the amygdala (planned comparison). No group by emotional category difference was found in the startle test. Increased harm avoidance and the observed changes in the 5-HT_1A_ receptor BP in the regions processing harm avoidance provides a plausible pathophysiological ground for the symptoms described in MCS, and yields valuable information for our general understanding of idiopathic environmental intolerances.

## Introduction

Multiple chemical sensitivity (MCS) is a common condition (prevalence of MCS is reported to be between 6% and 33% [1], [2], characterized by somatic distress upon exposure to odors [3], [4]. As in other idiopathic environmental intolerances the mechanisms behind the reported hypersensitivity are unknown. The estimated prevalence in population studies varies partly due to the chosen criteria as can be illustrated by the Danish study by Berg and coworkers [5]. In a random sample of 18 to 69 years old women and men 27% of the 4,242 respondents reported symptoms related to inhalation of airborne chemicals but only 0.5% had made adjustments due to this problem in their social as well as occupational life. Symptoms are triggered at exposure levels well below current exposure limits based on identified health risks. The triggering substances are chemically unrelated, indicating that no specific toxicological pathway may explain the reported reactions. In many cases the afflicted individuals suffer greatly from their odor intolerance and are unable to work or take part in everyday social life due to extensive avoidance behavior [6], [7]. MCS is more commonly reported in women than in men [1].

In our previous positron emission tomography (PET) activation study [8] we observed that subjects with MCS process odors differently from controls. MCS subjects were found to activate regions engaged in odor processing, (the amygdala, piriform cortex and the insular cortex) less than controls, and displayed an increased activation of the anterior cingulate cortex (ACC) and the cuneus-precuneus. Notably, the base-line rCBF (regional Cerebral Blood Flow) was not different from controls. As a possible explanation to these findings we suggested that MCS subjects could have enhanced top-down regulation of odor-response due to an increase in avoidant personality trait (harm avoidance).

Top-down modulation of emotional stimuli, as well as harm avoidance, is processed by the medial prefrontal cortex (mPFC), the ACC, and the amygdala [9], [10], [11]. The ACC is said to be primarily involved in signaling the presence of conflict, [12], [13] and may, thus, very well show an enhanced activation when subjects with MCS condition were exposed to odors. Using measurements of cortical surface with MRI in relation to the scores on a Temperament and Character Inventory (TCI) Pujol et al. found that surface area of the right ACC accounted for 24% score variance in Harm Avoidance [14]. In an fMRI study of stop inhibition task Yang and coworkers reported greater activation of the subgenual ACC in people with high levels of harm avoidance as compared to those with low [15], and Kim et al. found that scores of harm avoidance were inversely correlated with the ACC concentration of glutamate, and positively correlated with its concentration of GABA [16]. Amygdala engagement in processing of harm avoidance is suggested by the positive correlation between harm avoidance scores and the amygdala volume [17], and the observation that neurotoxic lesions of the amygdala reduce the harm avoidant behavior in Rhesus Monkeys [18].

Both the amygdala and the ACC express high densities of the serotonin 5-HT_1A_ receptor [19], [20]. The 5-HT_1A_ receptor binding is reported to be reduced in conditions associated with harm avoidance, such as anxiety and depression [21], [22]. Clinical studies of MCS using structured clinical interviews for DSM –IV disorders (SCID) reported a comorbidity between MCS and both these conditions [23]. Patients with MCS were also shown to score significantly higher on anxiety traits/neuroticism personality dimensions when compared to referents [24]. There are also several case reports indicating that antidepressants targeting the serotonergic system might be effective in the treatment of MCS [25], [26]. Taken together, these data strongly argue for an engagement of the serotonin system in the pathophysiology of both MCS and harm avoidance.

The 5-HT_1A_ is the major inhibitory serotonergic receptor on glutaminergic and gammaaminobutyric acid (GABA)ergic neurons in the frontal cortex [27], [28], [29]. Therefore, the inhibitory 5-HT_1A_ receptors strongly influence the effects of serotonergic firing on glutaminergic and GABAergic neurons in the frontal cortex and in the amygdala. Considering that the GABA levels and inhibitory impulses from the prefrontal cortex have been reported to be increased in harm avoidance we expected the frontal 5-HT_1A_ receptors to be down-regulated. As an effect of the presumably elevated top-down regulation of the amygdala from the ACC we hypothesized that the 5-HT_1A_ receptor binding would be down-regulated also.

Harm avoidance is associated with an abnormal startle reflex [30]. Like harm avoidance, the startle reflex is linked to ACC and amygdala function. In a PET study of brain correlates of startle modulation, a function reflecting the interaction between startle and affect, Pissiota and coworkers found, as a result of startle potentiation, a significant increase of rCBF medially in the affective division of ACC, and in a region covering the left amygdala-and hippocampus [30]. Thus, the startle reflex seems to involve similar circuits as those believed to process harm avoidance. This is of interest when discussing the pathophysiology of MCS, and raises the question as to whether the startle reflex under emotional influence could be abnormal (enhanced) in persons reporting MCS.

Taking into consideration all the aforementioned data, we designed a study combining PET and behavioral experiments in which we set out to test the hypotheses that MCS subjects show increased harm avoidance scores, and higher scores on personality traits of anxiety. We further hypothesized that MCS persons would have changes in the 5-HT_1A_ receptor binding, particularly in regions reported to be engaged in harm avoidance (primarily the ACC), and that their scores of trait harm avoidance would be related to the 5-HT_1A_ receptor binding potential (BP) in these regions. Considering our previous finding of a reduced odor activation of the amygdala in MCS persons, and the presumed enhancement of the top down modulation of the amygdala, we also expected that the BP would be altered in this region. In addition, we investigated whether the acoustic startle response to affective pictures would be altered in MCS relative controls.

## Results

### Psychological Profiles

**Figure 1:**
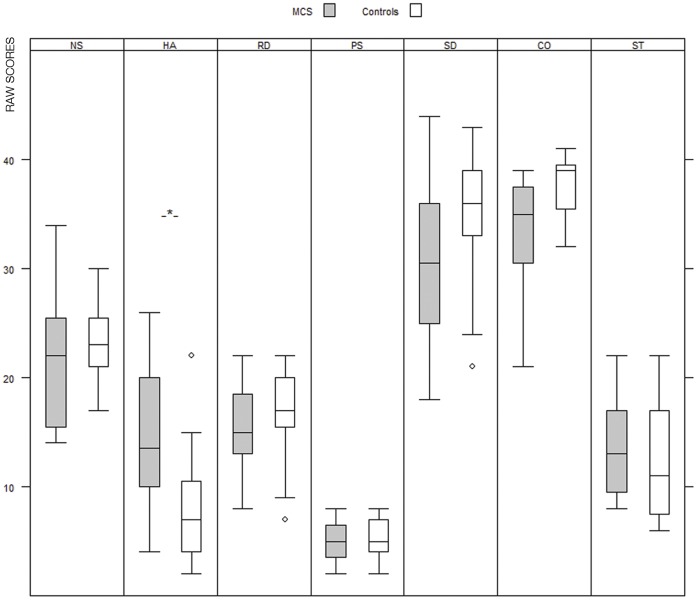
Personality dimensions in TCI (raw scores) in MCS subjects and controls (Boxes represent first quartile, median and third quartile, whiskers represent values within 1.5 interquartile range, a circle is an outlier value more than 1.5 IQR from the end of the box). Temperament dimensions: novelty seeking (NS), harm avoidance (HA), reward dependence (RD), and persistence (PS). Character dimensions: self-directedness (SD), cooperativeness (CO), self-transcendence (ST). *Hypothesis testing: one-sided p value, significance level 0.05 (HA = 0.01).

**Figure 2:**
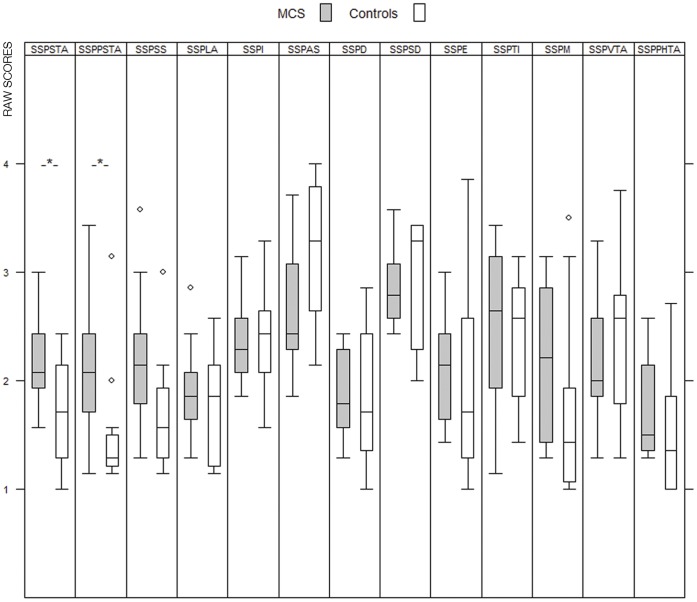
SSP raw scores in MCS subjects and controls (Boxes represent first quartile, median and third quartile, whiskers represent values within 1.5 interquartile range (IQR), a circle is an outlier value more than 1.5 IQR from the end of the box). Scales: Somatic trait anxiety (SSPSTA), Psychic trait anxiety (SSPPSTA), Stress susceptibility (SSPSS), Lack of assertiveness (SSPLA), Impulsiveness (SSPI), Adventure seeking (SSPAS), Detachment (SSPD), Social desirability (SSPSD), Embitterment (SSPE), Trait irritability (SSPTI), Mistrust (SSPM), Verbal trait aggression (SSPVTA) and Physical trait aggression (SSPPHTA). *Hypothesis testing: one-sided p value, significance level 0.05 (SSPSTA p = 0.04, SSPPSTA p = 0.006).

As hypothesized, the MCS subjects scored significantly higher in harm avoidance (p = 0.01 in one tailed test of specified hypothesis) in comparison to the control group (Figure 1). None of the other temperament or character dimensions in TCI differed significantly between the groups when the Bonferroni correction was applied. In addition, group differences supporting our hypotheses were detected in SSP scales with MCS persons rating higher in somatic and psychic trait anxiety (p = 0.04 and p = 0.006 respectively) in one-sided tests (Figure 2). There were no significant differences between the groups in any of the other scales after Bonferroni correction. Interestingly, MCS persons MADRS (included in the screening questionnaires) scores were below 2 (normal range 0–12) and were comparable to the scores of the controls (0–3).

### PET Data

**Figure 3:**
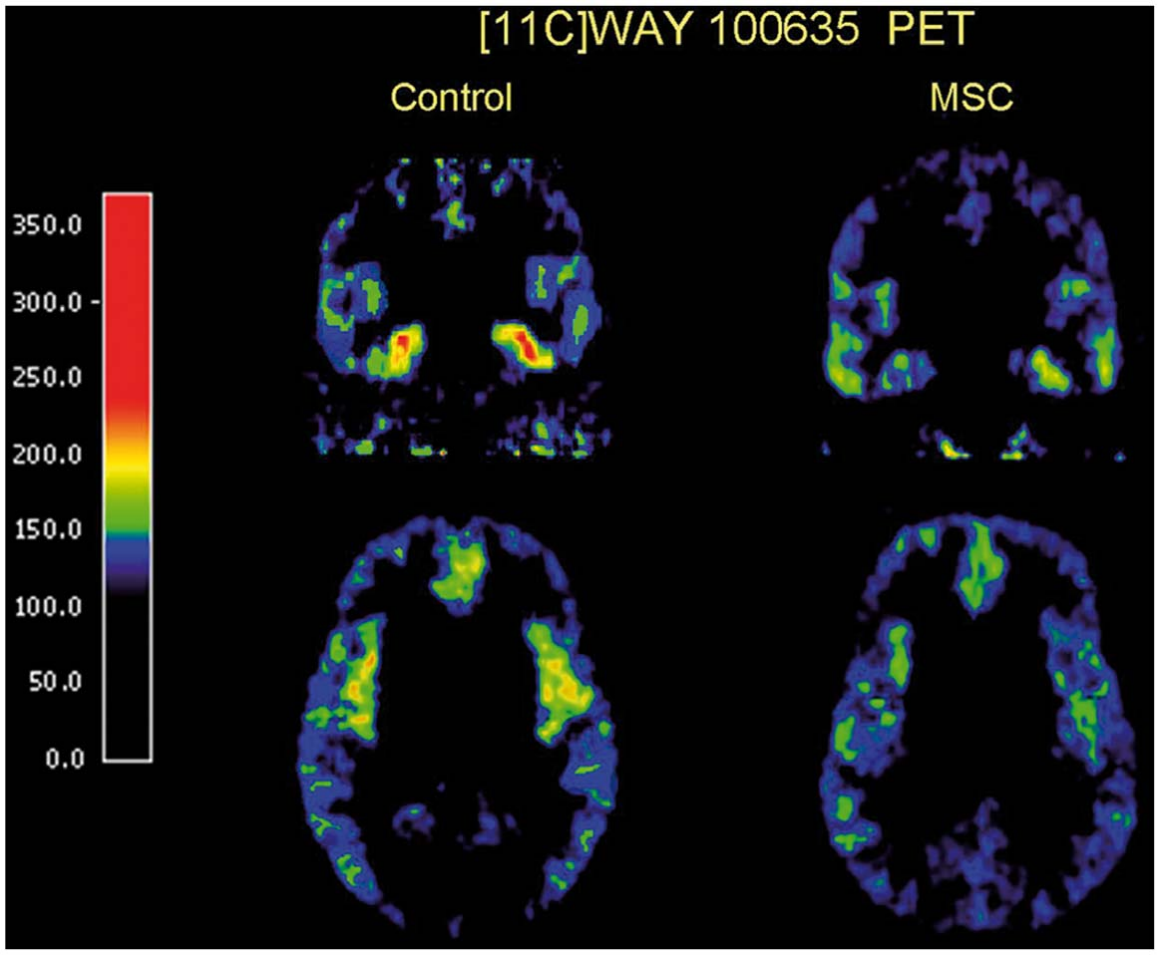
Summated positron emission tomography (PET) images representing average regional uptake of [11C] WAY100635 15–63 minutes after i.v. injection of the ligand in a control woman (left) and a MCS woman (right). The PET images show lower [11C] WAY100635 uptake in regions of amygdale (upper raw), anterior cingulate and insular cortex (lower raw) in MCS woman compared to control woman. The scale (nCi/cc) applies to both subjects, and the color coding was adjusted for the difference in injected radioactivity.

The results showed that MCS subjects differ from healthy controls with respect to the serotonin system. MCS subjects exhibited a significantly lower 5-HT_1A_ receptor binding potential in the amygdala and the anterior cingulate cortex (p = 0.029 and p = 0.005 respectively; planned comparisons, one tailed test of specified hypothesis, no correction for multiple comparisons, significance level 0.05) (Table 1, Figure 3). Numerically MCS subjects displayed lower binding potential also in other areas but the difference from healthy controls only passed the level of significance after the Bonferroni correction in the insular cortex (p = 0.003, significance level 0.005 with Bonferroni correction (Table 1)). As a complement to the non-parametric tests we also analyzed differences in binding effects across all regions by applying a repeated measures general linear model (GLM) with group as between subject factor and regions as within subject factors. The results showed a significant group effect (F(1,20) = 7.659, P = 0.012, partial eta squared = 0.277). There was also a significant interaction effect between group and region, F(3.293, 65.870) = 3.201, P = 0.025, partial eta squared = 0.138.

**Table 1:** PET 5-HT_1A_ receptor binding potential (15 frames) in MCS subjects and controls.

	MCS	Controls	*p*
Amygdala	4.87	5.99	**0.029** *
Anterior cingulate cortex	3.65	5.00	**0.005** *
Dorsolateral prefrontal cortex	3.07	4.03	0.07†
Hippocampus	4.56	6.84^1^	0.005† 1
Insular cortex	4.36	6.30	**0.003** †
Orbifrontal cortex	2.96	3.66	0.04†
Parietal cortex	3.25	4.03	0.05†
Temporal cortex	3.78	4.72	0.02†
Dorsal Raphe	2.76	3.38	0.02†
Medial prefrontal cortex	3.12	3.91	0.03†

(Median; Mann Whitney U-test).

1 *One control subject excluded due to extreme outlier.*

* *Hypothesis testing: one-sided p value, significance level 0.05.*

† *Significance level 0.005 with Bonferroni correction.*

The distribution of [11C]WAY 100635 in the reference region measured as the area under the curve/injected radioactivity * 100, did not differ between MCS and controls (2.52±0.90 nCi/cm3/min in MCS, 2.79±0.70 nCi/cm3/min in controls; p = 0.4).

### Startle Test

**Figure 4:**
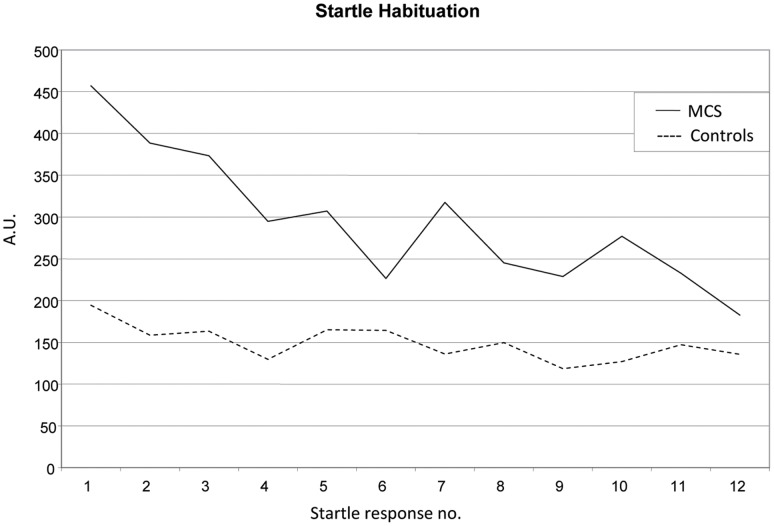
Startle magnitude (mean) during habituation before start of test during picture display. Habituation is more pronounced in the MCS group (p = 0.006). *A.U. =  Arbitrary units.*

Startle magnitudes decreased as a function of repeated exposure to the startle probe during habituation (Figure 4). Startle habituation was more pronounced in MCS subjects than controls (p = 0.006; Mann Whitney U-test of the linear trend). There was, however, no group by valence interaction when startle responses to neutral, positive and negative pictures were analyzed. The displayed pictures evoked different emotional reactions, in line with the intention of the test. Accordingly, both arousal and valence ratings of the displayed pictures differed significantly between valence categories (negative, neutral and positive). It was noted that MCS subjects displayed a general tendency to rate all pictures as more negative than controls (p = 0.03 for the between group comparison of ratings of all pictures).

### Correlation Analyses

We investigated whether there was any correlation between the 5-HT_1A_ receptor BP in the regions of interest (the amygdala and ACC) and variables in the psychological profiles for which differences between MCS and control subjects were observed. The latter included as reported above harm avoidance, somatic and psychic trait anxiety. No significant covariation was detected for the scores of harm avoidance and 5-HT_1A_ receptor BP in amygdale or ACC, neither in the MCS nor the control group. With regard to the SSP scales there was an inverse correlation in the MCS group regarding somatic trait anxiety and 5-HT_1A_ receptor BP in amygdale (p = 0.04 without Bonferroni correction as this was hypothesized, correlation coefficient −0.59). No correlation was detected between the insular BP and the harm avoidance or anxiety.

## Discussion

The present study shows that MCS persons have increased harm avoidance, and also specific reductions in the 5-HT_1A_ receptor BP in the amygdala, the ACC and the insular cortex (Figure 3). During the startle test MCS persons rated all pictures, regardless of emotional category, as more negative than the control subjects, and displayed more pronounced amplitude at the onset of startle, resulting in a steeper habituation curve relative to controls.

The results, thus, support the specified hypotheses tested, and imply that changes in the serotonin system may provide a physiological ground for the increase in harm avoidance and, perhaps in top-down modulation of the response to odor stimuli. While not directly hypothesized, also the observed changes in the insular cortex adhere well with our suppositions, considering that the insular cortex is involved in responses to disgust, and unpleasant somatosensory or emotional stimuli [31], [32]. Together, these observations advance our current information about this unexplained, yet common condition by emphasizing that persons with MCS have stationary cerebral changes rather than just an instant enhanced reaction to odors. Whilst such a possibility has long been discussed among the professionals, it has been difficult to verify with objective tests. Furthermore, studies focusing on possible differences in odor independent brain functioning in MCS subjects as compared to controls have to the large part not been hypotheses driven but more of an explorative kind or case reports [33], [34], [35]. Results have differed from study to study and chance findings cannot be ruled out. One recent SPECT study by Orriols and co-workers found decreased perfusion (before chemical challenge) in MCS subjects compared with controls in small cortical areas bilaterally in the temporal and fronto-orbital cortex and in the right parietal cortex [36].

MCS subjects in the present study scored higher in TCI for harm avoidance than the control group, and exhibited reductions in 5-HT_1A_ receptor BP in the amygdala, ACC and insular cortex. Even though no direct correlation was detected with harm avoidance these findings support our hypothesis of an altered serotonergic transmission in the central regions for top-down modulation of emotional and odor stimuli, and accord with previous notions that serotonin system is associated with harm avoidance (see e.g. [21], [37]–[40]). Our MCS subjects scored high also on trait anxiety, and their scorings correlated with low 5-HT_1A_ receptor BP in the amygdala. Higher scores on trait anxiety imply a higher degree of worrying in MCS subjects [41]. The results are congruent with the elevated harm avoidance in these subjects and provide additional argument for an involvement of the serotonin system in MCS. Personality traits have been suggested to be presented in a subclinical way before the onset of symptomatic conditions such as e.g. anxiety disorder [42]. Harm avoidance is associated with inhibited behaviors in relation to new stimuli [43] and predispose to fear-avoidance behavior [44]. Taken together, this makes harm avoidance a very plausible etiological factor in MCS.

The more pronounced habituation to the startle stimuli (noise) during the initial habituation tests is interesting and somewhat contradictory to what might be expected. The lack of indications of sensitization to this stimulus may be due to the fact that the sensitivity is limited to odors or due other factors as e.g. the time aspect. The 12 startle probes during the habituation phase sere presented with an inter-stimulus-interval of 12±3 s, i.e. a very short time period. A study by Dalton investigated a possible top-down regulation on perceived intensity of an odor [45]. Information presented prior to the exposure with the aim to introduce positive or negative bias to the odor was shown to influence the reported intensity but only after about 10 minutes. Initially all groups displayed a habituation to the odor. It would be of interest to further investigate the time factor regarding habituation to different stimuli in MCS subjects.

The GLM analyses revealed a general difference in binding potential between MCS and control subjects, but there was also a significant interaction between group and regions indicating that the differences between the groups were not the same in all regions. Our a priori hypotheses are supported in the non-parametric tests of differences between the groups in the respective region. These hypotheses were based on results in earlier studies by our group and associations between harm avoidance and deviations in the serotonergic system in specific regions in studies by other groups. There may possibly be also more general defenses between MCS subjects and controls but the relevance of differences in binding potentials in other areas with regard to the clinical picture of MCS subjects is not known. The reductions in receptor binding measured with PET may, theoretically, reflect changes in receptor density or affinity, or receptor down regulation, internalization or destruction, or blockage by the endogenous ligand even though [^11^C] WAY 100635 seems insensitive to endogenous levels of serotonin [46]. Therefore, any statement concerning the precise mechanisms underlying the present 5-HT_1A_ receptor BP reductions must be speculative. Serotonin 1_A_ receptors operate both as somatodendritic autoreceptors and as postsynaptic receptors. Somatodendritic 5-HT_1A_ autoreceptors are predominantly located on serotonin neurons and dendrites in the brainstem raphe complex. Their activation by serotonin or 5-HT_1A_ agonists decreases the firing rate of serotonergic neurons and subsequently reduces the synthesis, turnover, and release of 5-HT from nerve terminals in projection areas [47]. Postsynaptic 5-HT_1A_ receptors are widely distributed in forebrain regions that receive serotonergic input, especially in the prefrontal cortex including the ACC. An activation of these receptors results in membrane hyperpolarization and decreased neuronal excitability. From this follows that reduction in 5-HT_1A_ receptor BP in ACC neurons could cause a reduced inhibition and increased excitation of these neurons, leading to enhancement of the downstream GABAergic inhibition of the amygdala. Such a scenario is compatible with the observed harm avoidance in MCS patients, as well as the described increase in ACC activation and decrease in the activation of the amygdala and piriform cortex during odor exposure [8]. Whether the presently observed BP reductions in the amygdala, ACC and the insular cortex is the primary cause of symptoms in MCS subjects is, however, uncertain. The data, nevertheless, deserve attention especially as they might illustrate a more widespread phenomenon among several groups with environmental intolerances. Increased fatigue and asthenia (subclasses of TCI harm avoidance) has been reported in persons suffering from health problems attributed to dental fillings [48], and limbic changes in 5-HT_1A_ receptor BP has been detected in subjects with chronic fatigue syndrome [49]. The importance of the aforementioned traits and receptor changes as risk factors for development of various idiopathic environmental intolerances needs, thus, to be further investigated.

### Methodological Issues

Some methodological issues deserve a comment. Although MCS subjects scored higher than controls in harm avoidance, no significant difference was observed in relation to the norm scores (Swedish women). One possibility for this discrepancy could be that the norm population may be contaminated with subjects scoring high on harm avoidance, and, perhaps, also having MCS. Swedish studies have e.g. reported 19% for odor intolerance with affective and behavioral consequences [50] and 17% for odor annoyance [51]. By excluding persons with odor annoyance from the control group in our study our two groups may be more contrasting with regard to harm avoidance than a comparison between MCS subjects and the general population.

There was no correlation between harm avoidance and the 5-HT _1A_ receptor BP in any of the regions investigated. Such a correlation could, however, be expected, at least in the ACC, considering that the 5-HT_1A_ receptor mediates inhibitory processes, and that an association between harm avoidance and activation of the subgenual ACC has been observed by others [15]. One possible explanation is that our study group was too small to detect such an association should it exist. Another is that our population was rather homogenous, reducing the chance to detect a regional correlation.

A further limitation of the study is the selection of study group, which has to rely on self-reported health disturbances. It is possible that the results would have been different had the study group had more pronounced symptoms and avoidance behavior. The MCS group scored rather high on the modified Chemical Odor Intolerance Index (mean 17 on a scale from 0 to 25) but all MCS persons were working or studying and thus had no extreme avoidance behavior. In our clinical practice we have seen many subjects that are unable to work or even take part in everyday life due to health complaints triggered by odors. Exposure to odors are difficult to avoid in our modern society, perfumes are used in washing detergents, shops use branding scents in order to influence the emotions and moods of the customers etc. The MCS subjects in our study avoided some odors that triggered symptoms but it did not restrict their lives to a large degree.

Finally, because this is a cross sectional study, it is impossible to be certain whether the detected changes are cause or effect of MCS.

In conclusion, MCS persons express increased harm avoidance and anxiety, even in absence of odor stimuli. We suggest that these traits are linked to changes in the serotonin signaling. They may represent a susceptibility factor for MCS, although theoretically, they could also be downstream effects of odor intolerance. It is possible that a similar scenario operates with other forms of idiopathic environmental intolerances, and assessing the level of anxiety and harm avoidance is potentially important in early identification of individuals at risk of developing persisting health problems due to environmental stressors.

## Materials and Methods

### Subjects

Subjects in the MCS group were recruited by an advertisement in a local newspaper. The inclusion criteria used in the study were based on the 1999 consensus report [5]. The participants were to have symptoms (which involved multiple organ systems) reproducibly triggered by exposure to low levels of multiple chemically unrelated and odorous chemicals. The condition had to be chronic (experienced for more than 1 year), but symptoms should improve or resolve with removal of the incitants. Potential participants were first screened in a telephone interview and the fulfillment of the inclusion criteria were then confirmed in a structured interview with open questions on symptoms, triggering factors, duration and avoidance at the pretest medical check-up.

A detailed clinical history, particularly occupational, was taken in all patients included in the study. They were asked whether, according to their own experience, there existed any substance(s) that might have triggered the syndrome. A brief self-report measurement of chemical odor tolerance, a modified version of the Chemical Odor Intolerance Index al. [52] was filled out by participants. It included questions on how often the subject was annoyed by paint, perfume, car exhaust, cigarette smoke and printing ink (the two latter exposures replaced pesticide and new carpeting which are very rarely reported as triggering factors in Sweden). Each item was scored on a five-point scale ranging from never to always. A physical examination, focused particularly on neurologic, systemic and airway diseases, was performed. The screening also included assessment of depression, scored with Montgomery Asberg Depression Scale, MADRS [53].

The PET study group consisted of 12 MCS females, age 22 to 44 (mean 32.3, SD 7.7 years) who met the criteria by the 1999 consensus report [5]. Seven MCS subjects reported some allergy (e.g. to pollen or furry animals) and one MCS subject was on medication due to hypothyreosis (and clinically euthyroid at the time of the study). There were no indications of the presence of other diseases in the medical work up. The MCS participants were all working or studying but actively avoiding exposure to odorous chemicals. The median duration of MCS was 15 years (range 3 to 29 years). The scores in the modified Chemical Odor Intolerance Index (range 0–25) ranged from 11 to 21 (mean score 17). Eleven control subjects, age 24 to 43 (mean 30.7, SD 5.7 years), were recruited from the general population (seven of them participated in another study of 5-HT_1A_ receptor BP) [19]. They were healthy according to medical history, physical examination and routine laboratory tests. They denied any history of past or present odor sensitivity. There were no significant differences between MCS subjects and control women with regard to demographic variables such as age and educational level. All subjects had regular menstrual cycles and were non-smokers.

None of the participants had a history of neurologic or psychiatric disease, none of them were on drug therapy, each of them denied substance abuse, and all were euthymic. In the screening for depression using MADRS, no total score above 2 was observed (seven MCS subjects had a Total score of 0, three of 1 and two subjects 2). None of controls had MADRS exceeding 3 (the normal range is 0–12). Subjects gave written informed consent and the study was approved by the Ethical Committee at the Karolinska Institutet and the Radiation Safety Committee of the Karolinska Hospital. The study was performed according to the guidelines of the Declaration of Helsinki 1975.

All MCS subjects and eight subjects from the PET control group were included in the startle test. Four subjects from the control group were unable to participate in the test (mainly because of new places of residencies). To compensate for this, four new control subjects were recruited for the startle test (all healthy females, startle control group age 24 to 44; mean 31.7, SD 6.5 years).

## Methods

### 

#### Psychological profiles

Two psychological inventories, the Temperament and Character Inventory (TCI) and the Swedish universities Scales of Personality (SSP), were used to characterize participants and more specifically assess harm avoidance and trait anxiety. The TCI is a 238 item, true-false, self-administered with the inventory being based on Cloninger’s personality theory [43]; [54]–[56]. The model encompasses four dimensions of temperament and three dimensions of character. Temperament refers to individual differences in conditioned emotional responses, such as anger, fear, and disgust. The temperament dimensions are traits, which are moderately heritable and moderately stable throughout life, namely, novelty seeking (NS), harm avoidance (HA), reward dependence (RD), and persistence (PS). Character refers to individual differences in goals, values, and self-conscious emotions such as shame, guilt, and empathy. The character dimensions are self-directedness (SD), cooperativeness (CO), and self-transcendence (ST). Thus, the character dimensions represent traits that are weakly heritable and moderately influenced by social learning; traits that can reach various levels of maturity in an incremental manner. A more detailed description of the dimensions is available elsewhere [43], [56]. Norms for the Swedish version of TCI was published by Brändström and coworkers [57].

The Swedish universities Scales of Personality (SSP) is a revised, shortened, modernized and psychometrically evaluated version of the Karolinska Scales of Personality (KSP) with a reduced number of items and improved psychometric quality [41]. It includes 91 items divided into 13 scales, i.e. Somatic trait anxiety, Psychic trait anxiety, Stress susceptibility, Lack of assertiveness, Impulsiveness, Adventure seeking, Detachment, Social desirability, Embitterment, Trait irritability, Mistrust, Verbal trait aggression and Physical trait aggression. In the present study the purpose of including these scales were to test the items that on the basis of the current literature could be expected to differ between persons reporting MCS and controls. Thus, TCI was used to test harm avoidance and SSP employed to investigate Somatic and Psychic trait anxiety.

#### Acoustic startle test

The startle reflex is triggered by intense and surprising stimuli and can be measured by recording the eye blink reflex [58]. The most commonly used stimulus is the acoustic stimulus consisting of a brief burst of white noise. Electromyography (EMG) registration of the right orbicularis oculi was performed using Psylab (Contact Precision Instruments inc., London, UK). Two Ag–AgCl electrodes filled with electrode gel were used for this purpose. One electrode was placed under the pupil and the other 1–2 cm lateral to the first [59]. The ground electrode was placed on the forehead. White noise with 100 ms duration and near instantaneous rise time, delivered binaurally through head phones, was used as startle probes to trigger the blink reflex. Intensity of the startle probe was gradually increased during a workup procedure until subjects reported the sound level to be tolerable but not painful. EMG data were collected using 100 Hz sampling rate. Data were low pass filtered at 500 Hz, high pass filtered at 30 Hz, and rectified prior to A/D conversion. Response amplitude was quantified as the maximum response in a time window 20–120 ms after stimulus onset subtracted from a baseline defined as the mean value 100–0 ms prior to stimulus onset. Startle blink responses were first recorded during a habituation phase where 12 startle probes were presented with an inter-stimulus-interval of 12±3 s. Immediately following the habituation phase, subjects viewed pictures of neutral, positive and negative valence on a computer monitor. Pictures were taken from the International Affective Picture System (IAPS) [60] and were displayed for 6 s. Twelve pictures were presented from each stimulus category (neutral, positive, negative) and a startle probe was delivered 5±0.8 s following picture onset. Mean normative ratings for women of the 3 stimulus categories were as following: neutral valence = 5.0±0.4, neutral arousal = 3.0±0.6, positive valence = 7.4±0.7, positive arousal = 5.7±1.1, negative valence = 2±0.8, negative arousal = 6.8±0.7 [60]. Response amplitudes were transformed to T-scores to reduce inter-individual variance and gain statistical power in the valence by group comparison [59] Responses from the habituation phase were not converted to T-scores but are reported in arbitrary units to allow for an evaluation of group differences in baseline startle response and habituation rate of startle. After the startle test, the subjects scored each shown picture with regard to positive and negative valence as well as the degree of arousal the picture evoked on a scale ranging from 1 to 9.

#### MRI

Structural images were acquired according to a previously described protocol [61] with a 1.5 Tesla Sigma 5.X scanner, (General Electric, Milwaukee, Wisconsin), and included 3D-weighted T1 SPGR images with 1-mm sections, used for measures of hippocampal volume, and the whole brain volumes (sum of the grey, white matter volume and cerebrospinal fluid), segmented with the SPM2 software package (Wellcome Department of Cognitive Neurology, London, http://www.fil.ion.ucl.ac.uk/spm)].

#### PET measurements of 5-HT_1A_ receptor binding potential

The binding potential (BP) to 5-HT_1A_ receptor was investigated using PET images acquired with an ECAT Exact HR 47 scanner (CTI/Siemens, Knoxville, TN) run in 3D mode (transaxial resolution of 3.8 mm), after bolus injection (190–267 MBq, 800–2000 mCi/cc) of [^11^C]WAY100635. Radioactivity in the brain was measured in a series of 15 consecutive frames for 63 minutes, of which the nine first frames were acquired over 15 minutes. Image processing included co-registration of MRI and sum PET images (representing the decay corrected average uptake of [^11^C]WAY-100635 during 15 to 63 minutes after ligand injection), and re-slicing of PET images to avoid spatial mismatch between the two modalities (SPM2). Three-dimensional volumes of interest (VOIs) were then delineated on original individual MR images and transferred to the corresponding individual PET images (first sum image, and dynamic image). The VOI analysis was preferred to an explorative evaluation of regional changes, because of the risk of a type II error, which was undesirable in this initial study of possible serotonin receptor changes in relation to MCS. As in our previous studies homologous VOIs were delineated from the hippocampus, amygdala, insular cortex, the temporal neocortex, the dorsolateral prefrontal cortex, the orbitofrontal and parietal cortex, the ACC, the raphe nuclei [20], cerebellum [19], and a mPFC VOI covering Brodmann Area 10 [62]. The three types of neocortical VOIs were included to test for possible 5-HT_1A_ changes outside the limbic networks. The cerebellar cortex (mean of both cerebellar VOIs excluding the cerebral vermis where 5-HT_1A_ receptors have been detected) [63] served as a reference region. Raphe nuclei were delineated because they contain the serotonergic cell bodies, although it is well known that these data have quiet poor validity because of partial volume effects. The raphe VOI covered both sides’ raphe nuclei. It was delineated directly on the sum PET images because raphe nuclei are inadequately visualized by MRI, but clearly visible on [^11^C]WAY 100 635 images. For detailed information on VOI definition see our previous publication [64].

Individual regional time-activity curves (TACs) generated during the 63 minute long scans were derived from each VOI. The BP was then estimated with simplified reference tissue model (SRTM) [65], [66]. As no significant asymmetries (p<0.05) were present between the right and left side, the mean BP was used as input for the statistical analyses. To ascertain that the cerebellar input function was similar in patients and controls, the cerebellar radioligand uptake was compared calculating the area under the curve (AUC)/injected radioactivity (p<0.05). Because BP varies considerably between various cerebral areas separate analyses of variance (ANOVA) were used for each VOI, factoring for subject group (p<0.005 with Bonferroni correction).

#### Statistical analyses

Statistical analyses were performed in IBM SPSS Statistics v20. Due to the small sample size we choose to use non-parametric tests as Mann-Whitney U-test and Spearman’s rank correlation coefficient. The Bonferroni correction was applied to correct for multiple testing and according to Bonferroni the significance level for the individual tests was set to 0.05 divided by the number of tests in order to maintain an overall significance level of 0.05. However, for hypotheses specifically defined and tested in our study we used one-tailed tests and did not apply the Bonferroni correction. The hypotheses based on results from our previous study [8] and the literature described above were:

that MCS persons would score higher in trait harm avoidance, somatic trait anxiety and psychic trait anxiety compared to controls
that MCS persons would show a reduced 5-HT_1A_ receptor binding potential in the amygdala and ACC
that regions showing changes in receptor BP would show correlations with traits which differed between MCS persons and controls
that MCS persons differ from controls in emotional modulation of acoustic startle response.

## References

[1] Kreutzer R, Neutra RR, Lashuay N (1999) Prevalence of people reporting sensitivities to chemicals in a population-based survey. Am J Epidemiol 150: 1–12. 10400546 10.1093/oxfordjournals.aje.a009908

[2] Meggs WJ, Dunn KA, Bloch RM, Goodman PE (1996) Prevalence and nature of allergy and chemical sensitivity in a general population. Arch Environ Health 51: 275–282. 8757407 10.1080/00039896.1996.9936026

[3] Berg ND, Linneberg A, Dirksen A, Elberling J (2008) Prevalence of self-reported symptoms and consequences related to inhalation of airborne chemicals in a Danish general population. Int Arch Occup Environ Health 81: 881–887. 18058120 10.1007/s00420-007-0282-0

[4] Cullen MR (1987) The worker with multiple chemical sensitivities. Occup Med 2: 655–661. 3313760

[5] Anonymous (1999) Multiple chemical sensitivity: a 1999 consensus. Arch Environ Health 54: 147–149. 10444033 10.1080/00039899909602251

[6] Lavergne MR, Cole DC, Kerr K, Marshall LM (2010) Functional impairment in chronic fatigue syndrome, fibromyalgia, and multiple chemical sensitivity. Can Fam Physician 56: e57–65. 20154232 PMC2821254

[7] Skovbjerg S, Brorson S, Rasmussen A, Johansen JD, Elberling J (2009) Impact of self-reported multiple chemical sensitivity on everyday life: a qualitative study. Scand J Public Health 37: 621–626. 19411319 10.1177/1403494809105430

[8] Hillert L, Musabasic V, Berglund H, Ciumas C, Savic I (2007) Odor processing in Multiple Chemical Sensitivity. Human Brain Mapping 28: 172–182. 16767766 10.1002/hbm.20266 PMC6871299

[9] Cisler JM, Koster EHW (2009) Mechanisms of attentional biases towards threat in anxiety disorders: An integrative review. Clinical Psychology Review 30: 203–216. 20005616 10.1016/j.cpr.2009.11.003 PMC2814889

[10] Enge S, Fleischhauer M, Lesch KP, Strobel A (2011) On the role of serotonin and effort in voluntary attention: evidence of genetic variation in N1 modulation. Behav Brain Res 216: 122–128. 20655956 10.1016/j.bbr.2010.07.021

[11] Sarter M, Gehring WJ, Kozak R (2006) More attention must be paid: the neurobiology of attentional effort. Brain Res Rev 51: 145–160. 16530842 10.1016/j.brainresrev.2005.11.002

[12] Aupperle RL, Paulus MP (2010) Neural systems underlying approach and avoidance in anxiety disorders. Dialogues Clin Neurosci 12: 517–531. 21319496 10.31887/DCNS.2010.12.4/raupperle PMC3181993

[13] Chiew KS, Braver TS (2011) Neural Circuitry of Emotional and Cognitive Conflict Revealed through Facial Expressions. PLoS ONE 6(3): e17635 doi:10.1371/journal.pone.0017635. 21408006 10.1371/journal.pone.0017635 PMC3052361

[14] Pujol J, Lopez A, Deus J, Cardoner N, Vallejo J, et al (2002) Anatomical variability of the anterior cingulate gyrus and basic dimensions of human personality. Neuroimage 15: 847–855. 11906225 10.1006/nimg.2001.1004

[15] Yang TT, Simmons AN, Matthews SC, Tapert SF, Frank GK, et al (2009) Adolescent subgenual anterior cingulate activity is related to harm avoidance. Neuroreport 20: 19–23. 19034055 10.1097/WNR.0b013e328317f3cb PMC2852645

[16] Kim HJ, Kim JE, Cho G, Song IC, Bae S, et al (2009) Associations between anterior cingulate cortex glutamate and gamma-aminobutyric acid concentrations and the harm avoidance temperament. Neurosci Lett. 464: 103–107. 10.1016/j.neulet.2009.07.087 19660524

[17] Iidaka T, Matsumoto A, Ozaki N, Suzuki T, Iwata N, et al (2006) Volume of left amygdala subregion predicted temperamental trait of harm avoidance in female young subjects. A voxel-based morphometry study. Brain Res 1125: 85–93. 17113049 10.1016/j.brainres.2006.09.015

[18] Machado CJ, Emery NJ, Mason WA, Amaral DG (2010) Selective changes in foraging behavior following bilateral neurotoxic amygdala lesions in rhesus monkeys. Behav Neurosci 124: 761–772. 21133532 10.1037/a0021560 PMC3034241

[19] Jovanovic H, Lundberg J, Karlsson P, Cerin Å, Saijo T, et al (2008) Sex differences in the serotonin 1A receptor and serotonin transporter binding in the human brain measured by PET. Neuroimage 39: 1408–1419. 18036835 10.1016/j.neuroimage.2007.10.016

[20] Savic I, Lindström P, Gulyás B, Halldin C, Andrée B, et al (2004) Limbic reductions of 5-HT1A receptor binding in human temporal lobe epilepsy. Neurology 62: 1343–1351. 15111672 10.1212/01.wnl.0000123696.98166.af

[21] Hansenne M, Pitchot W, Moreno AG, Reggers J, Machurot PY, et al (1997) Harm avoidance dimension of the Tridimensional Personality Questionnaire and serotonin-1A activity in depressed patients. Biol Psychiatry 42: 959–961. 9359984 10.1016/s0006-3223(97)00325-9

[22] Lanzenberger RR, Mitterhauser M, Spindelegger C, Wadsak W, Klein N, et al (2007) Reduced serotonin-1A receptor binding in social anxiety disorder. Biol Psychiatry 61: 1081–1089. 16979141 10.1016/j.biopsych.2006.05.022

[23] Hausteiner C, Bornschein S, Bickel H, Zilker T, Forstl H (2003) Psychiatric morbidity and low self-attentiveness in patients with environmental illness. J Nerv Ment Dis 191: 50–55. 12544600 10.1097/00005053-200301000-00009

[24] Osterberg K, Persson R, Karlson B, Carlsson Eek F, Orbaek P (2007) Personality, mental distress, and subjective health complaints among persons with environmental annoyance. Hum Exp Toxicol 26: 231–241. 17439926 10.1177/0960327107070575

[25] Ronnback AP, Jarvholm B (1997) Successful use of a selective serotonin reuptake inhibitor in a patient with multiple chemical sensitivities. Acta Psychiatr Scand 96: 82–83. 9259230 10.1111/j.1600-0447.1997.tb09910.x

[26] Stenn P, Binkley K (1998) Successful Outcome in a Patient With Chemical Sensitivity. Psychosomatics 39: 547–550. 9819957 10.1016/S0033-3182(98)71289-7

[27] Amargós-Bosch M, Bortolozzi A, Puig MV, Serrats J, Adell A, et al (2004) Co-expression and in vivo interaction of serotonin1A and serotonin2A receptors in pyramidal neurons of prefrontal cortex. Cereb Cortex 14: 281–299. 14754868 10.1093/cercor/bhg128

[28] Puig MV, Artigas F, Celada P (2005) Modulation of the activity of pyramidal neurons in rat prefrontal cortex by raphe stimulation in vivo: involvement of serotonin and GABA. 10.1093/cercor/bhh104 15238448

[29] Cereb Cortex. 15: 1–14. 10.1093/cercor/bhh104 15238448

[30] Santana N, Bortolozzi A, Serrats J, Mengod G, Artigas F (2004) Expression of serotonin1A and serotonin2A receptors in pyramidal and GABAergic neurons of the rat prefrontal cortex. Cereb Cortex 14: 1100–1109. 15115744 10.1093/cercor/bhh070

[31] Pissiota A, Frans O, Michelgård A, Appel L, Långström B, et al (2003) Amygdala and anterior cingulate cortex activation during affective startle modulation: a PET study of fear. Eur J Neurosci 18: 1325–1331. 12956731 10.1046/j.1460-9568.2003.02855.x

[32] Jabbi M, Bastiaansen J, Keysers C (2008) A common anterior insula representation of disgust observation, experience and imagination shows divergent functional connectivity pathways. PLoS One 3(8): e2939 doi:10.1314/journal.pone.0002939. 18698355 10.1371/journal.pone.0002939 PMC2491556

[33] Mohr C, Leyendecker S, Mangels I, Machner B, Sander T, et al (2008) Central representation of cold-evoked pain relief in capsaicin induced pain: an event-related fMRI study. Pain 139: 416–430. 18614290 10.1016/j.pain.2008.05.020

[34] Heuser G, Mena I, Alamos F (1994) NeuroSPECT findings in patients exposed to neurotoxic chemicals. Toxicol Ind Health 10: 561–571. 7778114

[35] Simon TR, Hickey DC, Fincher CE, Johnson AR, Ross GH, Rea WJ (1994) Single photon emission computed tomography of the brain in patients with chemical sensitivities. Toxicol Ind Health 10: 573–577. 7778115

[36] Ross GH, Rea WJ, Johnson AR, Hickey DC, Simon TR (1999) Neurotoxicity in single photon emission computed tomography brain scans of patients reporting chemical sensitivities. Toxicol Ind Health 15: 415–420. 10416294 10.1177/074823379901500316

[37] Orriols R, Costa R, Cuberas G, Jacas C, Castell J, et al (2009) Brain dysfunction in multiple chemical sensitivity. J Neurol Sci 287: 72–78. 19801154 10.1016/j.jns.2009.09.003

[38] Jacob CP, Strobel A, Hohenberger K, Ringel T, Gutknecht L, et al (2004) Association between allelic variation of serotonin transporter function and neuroticism in anxious cluster C personality disorders. Am J Psychiatry 161: 569–572. 14992987 10.1176/appi.ajp.161.3.569

[39] Peirson AR, Heuchert JW, Thomala L, Berk M, Plein H, et al (1999) Relationship between serotonin and the temperament and character inventory. Psychiatry Res 89: 29–37. 10643875 10.1016/s0165-1781(99)00079-7

[40] Strobel A, Gutknecht L, Rothe C, Reif A, Mossner R, et al (2003) Allelic variation in 5-HT(1A) receptor expression is associated with anxiety- and depression-related personality traits. J Neural Transm 110: 1445–1453. 14666415 10.1007/s00702-003-0072-0

[41] Wiesbeck GA, Weijers HG, Wodarz N, Keller HK, Michel TM, et al (2004) Serotonin transporter gene polymorphism and personality traits in primary alcohol dependence. World J Biol Psychiatry 5: 45–48. 15048635 10.1080/15622970410029907

[42] Gustavsson JP, Bergman H, Edman G, Ekselius L, von Knorring L, et al (2000) Swedish universities Scales of Personality (SSP): construction, internal consistency and normative data. Acta Psychiatr Scand 102: 217–225. 11008858 10.1034/j.1600-0447.2000.102003217.x

[43] Brandes M, Bienvenu OJ (2006) Personality and anxiety disorders. Curr Psychiatry Rep 8: 263–269. 16879789 10.1007/s11920-006-0061-8

[44] Cloninger CR, Svrakic DM, Przybeck TR (1993) A psychobiological model of temperament and character. Arch Gen Psychiatry 50: 975–990. 8250684 10.1001/archpsyc.1993.01820240059008

[45] Conrad R, Schilling G, Bausch C, Nadstawek J, Wartenberg HC (2007) Temperament and character personality profiles and personality disorders in chronic pain patients. Pain 133: 197–209. 17964076 10.1016/j.pain.2007.07.024

[46] Dalton P (1996) Odor perception and beliefs about risk. Chem Senses 21: 447–458. 8866108 10.1093/chemse/21.4.447

[47] Rabiner EA, Messa C, Sargent PA, Husted-Kjaer K, Montgomery A, et al (2002) A database of [(11)C]WAY-100635 binding to 5-HT(1A) receptors in normal male volunteers: normative data and relationship to methodological, demographic, physiological, and behavioral variables. Neuroimage 15: 620–632. 11848705 10.1006/nimg.2001.0984

[48] Hamon M, Lanfumey L, el Mestikawy S, Boni C, Miquel MC, et al (1990) The main features of central 5-HT1 receptors. Neuropsychopharmacology 3: 349–360. 2078271

[49] Bergdahl J, Mårell L, Bergdahl M, Perris H (2005) Psychobiological personality dimensions in two environmental-illness patient groups. Clin Oral Investig 9: 251–256. 10.1007/s00784-005-0015-2 16215748

[50] Cleare AJ, Messa C, Rabiner EA, Grasby PM (2005) Brain 5-HT1A receptor binding in chronic fatigue syndrome measured using positron emission tomography and [11C]WAY-100635. Biol Psychiatry 57: 239–246. 15691524 10.1016/j.biopsych.2004.10.031

[51] Johansson A, Brämerson A, Millqvist E, Nordin S, Bende M (2005) Prevalence and risk factors for self-reported odour intolerance: the Skövde population-based study. Int Arch Occup Environ Health 78: 559–564. 16001204 10.1007/s00420-005-0616-8

[52] Carlsson F, Karlson B, Ørbaek P, Osterberg K, Ostergren PO (2005) Prevalence of annoyance attributed to electrical equipment and smells in a Swedish population, and relationship with subjective health and daily functioning. Public Health 119: 568–577. 15925670 10.1016/j.puhe.2004.07.011

[53] Szarek MJ, Bell IR, Schwartz GE (1997) Validation of a brief screening measure of environmental chemical sensitivity: the chemical odor intolerance index. J Environ Psychol 17: 345–351.

[54] Montgomery SA, Åsberg M (1979) A new depression scale designed to be sensitive to change. Br J Psych 134: 322–389. 10.1192/bjp.134.4.382 444788

[55] Cloninger CR, Gilligan SB (1987) Neurogenetic mechanisms of learning: a phylogenetic perspective. J Psychiatr Res 21: 457–472. 3326938 10.1016/0022-3956(87)90094-x

[56] Cloninger CR, Przybeck TR, Svrakic DM (1991) The Tridimensional Personality Questionnaire: U.S. normative data. Psychol Rep 69: 1047–1057. 1784653 10.2466/pr0.1991.69.3.1047

[57] Cloninger CR, Przybeck TR, Svrakic DM, Wetzel RD (1994) The Temperament and Character Inventory (TCI). A Guide to Its Development and Use. St. Louis, MO: Center for Psychobiology of Personality, Washington University.

[58] Brändström S, Schlette P, Przybeck TR, Lundberg M, Forsgren T, et al (1998) Swedish normative data on personality using the Temperament and Character Inventory. Compr Psychiatry 39: 122–128. 9606577 10.1016/s0010-440x(98)90070-0

[59] Lang PJ, Bradley MM, Cuthbert BN (1990) Emotion, Attention and the Startle Reflex. Psychol Rev 97: 377–395. 2200076

[60] Blumenthal TD, Cuthbert BN, Filion DL, Hackley S, Lipp OV, et al (2005) Committee report: Guidelines for human startle eyeblink electromyographic studies. Psychophysiol 42: 1–15. 10.1111/j.1469-8986.2005.00271.x 15720576

[61] Lang PJ, Bradley MM, Cuthbert BN (1997) International Affective Picture System (IAPS): Technical Manual and Affective Ratings. NIMH Center for the Study of Emotion and Attention.

[62] Ciumas C, Savic I (2006) Structural changes in patients with primary generalized tonic and clonic seizures. Neurology 67: 683–686. 16924024 10.1212/01.wnl.0000230171.23913.cf

[63] Ochsner KN, Zaki J, Hanelin J, Ludlow DH, Knierim K, et al (2008) Your pain or mine? Common and distinct neural systems supporting the perception of pain in self and other. Soc Cogn Affect Neurosci 3: 144–160. 19015105 10.1093/scan/nsn006 PMC2555461

[64] Slater P, Doyle CA, Deakin JF (1998) Abnormal persistence of cerebellar serotonin-1A receptors in schizophrenia suggests failure to regress in neonates. J Neural Transm 105: 305–315. 9660109 10.1007/s007020050060

[65] Jovanovic H, Perski A, Berglund H, Savic I (2011) Chronic stress is linked to 5-HT(1A) receptor changes and functional disintegration of the limbic networks. Neuroimage 55: 1178–1188. 21211567 10.1016/j.neuroimage.2010.12.060

[66] Lammertsma AA, Hume SP (1996) Simplified reference tissue model for PET receptor studies. Neuroimage 4: 153–158. 9345505 10.1006/nimg.1996.0066

[67] Gunn RN, Lammertsma AA, Hume SP, Cunningham VJ (1997) Parametric imaging of ligand-receptor binding in PET using a simplified reference region model. Neuroimage 6: 279–287. 9417971 10.1006/nimg.1997.0303

